# Peripheral Placentitis

**Published:** 1873-03-15

**Authors:** H. Wardner

**Affiliations:** Cairo, Ill.


### PERIPHERAL PLACENTITIS.

BY H. WARDNER, M.D., CAIRO, ILL.

In the month of July, 1872, my attention was called to the case of a lady who had been suffering some eight or nine weeks from a discharge from the uterus, of a hæmorrhagic character, which had resisted the usual remedies in like cases.

The *os uteri* presented a granular, spongy appearance, with eversion of a congested mucous membrane, that would bleed on the slightest touch with the probe. The uterus was enlarged to about the size usual between the second and third months of pregnancy; but whether it contained a tumor or a fœtus, it was difficult, at the time, to determine. The patient, however, was positive she was not pregnant.

I applied the fluid extract of pinus Canadensis, on a pledget of cotton, which seemed to control the discharge for a couple of days; and then it returned as before. The application was repeated in five days, and gallic acid given internally, but without the relief sought. On the third day after the second application, she passed a discolored fœtus, apparently of the second or third month, which had evidently been dead some time. About one hour after, I removed the placenta, which presented an appearance so peculiar as to attract particular attention. It was thickened and cup-shaped, with a very dark, shreddy appearance of the entire periphery, extending about one inch from the margin back along the uterine surface. The evidences of inflammatory action were unmistakable; and my impression, at the time, was that there had been a gradual detachment from the uterine surface around its entire margin, thus causing the "flow," which was changeable in its character from red blood and small clots to a dark-colored fluid, with occasional shreds of degenerated tissue. None of the remedies used in this case had any control over the discharge, except temporarily, and no relief came to the patient until the discharge of the fœtus, after which she recovered her usual health in due process of time.

I found nothing in the works on Obstetrics on similar diseases of the placenta; but, on looking over the "Half-Yearly Compendium" (No. X, page 203), I saw a report of a similar case, made by Dr. Rogers, to the New York Pathological Society, which was pronounced by Dr. Neggerath to be a case of "*Peripheral Placentitis.*"

The disease is of such very rare occurrence that I thought this case might not be unworthy of record in medical literature.

The Fate of Empires.—M. Piorry has written to the *Academie de Medecine* to express regret that, prior to the Franco-German war, the size and shape of the kidneys in the late Emperor Napoleon III. were not investigated by "plessimetric percussion." A little more plessimetry, and France might have been saved. On such threads does the fate of empires hang!

Steps are being taken to encourage the study of ophthalmology in France.

